# Competing risk nomogram predicting cause-specific mortality in older patients with testicular germ cell tumors

**DOI:** 10.3389/fmed.2024.1327485

**Published:** 2024-04-17

**Authors:** Xiaoying Wu, Mingfei Zhou, Jun Lyu, Lin Chen

**Affiliations:** ^1^College of Pharmacy, Jinan University, Guangzhou, China; ^2^Drug Clinical Trial Institution, The First Affiliated Hospital of Jinan University, Guangzhou, China; ^3^Department of Clinical Research, The First Affiliated Hospital of Jinan University, Guangzhou, China

**Keywords:** competing risk, older patients, testicular germ cell tumors, nomogram, prognosis

## Abstract

**Background:**

Testicular germ cell tumor (TGCT) is the most common type of malignancy in young men, but rarely in older adults. We aimed to construct a competing risk model to predict the prognosis for older patients with TGCT.

**Methods:**

We collected TGCT patients aged 50 years or older diagnosed between 2004 and 2015 from the National Cancer Institute’s Surveillance, Epidemiology, and End Results (SEER) database. We estimated the cumulative incidences of cause-specific death (CSD) and other causes of death and established a nomogram predicting cause-specific mortality in older patients with TGCT by Fine-Gray competing risk regression. The concordance index (C-index), calibration curves, area under the receiver operating characteristic curve (AUC), and decision analysis curves (DCA) were used to evaluate the differentiation, accuracy, and clinical significance of the nomogram.

**Results:**

A total of 2,751 older TGCT patients were included in the study. The 3-, 5-, and 10-year cumulative incidences were 4.4, 5.0 and 6.1%, respectively, for cause-specific death, and 3.8, 6.2, 13.1%, respectively, for other causes of death. Predictors of cause-specific mortality in older TGCT included age, marital status, annual household income, histology, tumor size, stage and surgery. In the training and validation sets, the C-indexes were greater than 0.8, indicating that the nomogram had good discrimination. The AUC revealed the same result. The calibration curves showed good agreement between the predicted and observed results of the nomogram. DCA curves indicated that the nomogram had more clinical significance than the conventional American Joint Committee on Cancer (AJCC) staging. Based on the total nomogram score of each case, all patients were categorized into low-risk and high-risk groups, and risk categorization allowed the identification of cases with a high risk of death.

**Conclusion:**

We established a competing risk nomogram with good performance that may help clinicians accurately predict the prognosis of older TGCT patients.

## Background

Testicular cancer is a relatively rare tumor, accounting for about 1% of newly diagnosed cancers in men worldwide. However, it is the most common malignant tumor in young men aged 14–44 years in Western countries (1). According to the American Cancer Society, there will be around 9,910 new cases of testicular cancer in the United States in 2021, with roughly 460 deaths from the disease (2). Over the last two decades, the global incidence of testicular cancer has grown (3). Testicular germ cell tumor (TGCT) is the most common tumor type among testicular malignancies, accounting for 95% of testicular malignancies, which are divided into seminomatous germ cell tumor (SGCT) and nonseminomatous germ cell tumor (NSGCT). The latter includes four main subtypes: embryonal carcinoma, choriocarcinoma, yolk sac tumor and teratoma, which are all more aggressive than the former (4). There is evidence of a significant age shift towards older age at diagnosis of germ cell tumors, with the average age increasing from 28 to 36 years (5). Several studies have been conducted to determine whether TGCT behave differently in younger and older people. Cancer-specific survival was found to be higher in younger individuals than in older patients (6, 7). This distinction is especially obvious in metastatic disease (8). More importantly, the 5-year survival rate for men diagnosed under the age of 50 is as high as 90%, but less than 70% for men aged 70–79 (6). As a result, precise prognostic prediction for older TGCT patients must be performed.

The AJCC staging system is widely used internationally to evaluate the staging of testicular tumor patients for subsequent therapy options and prognosis assessment (9). However, the AJCC staging system primarily considers anatomical characteristics of the tumor and disregards additional prognostic markers like age and histologic type (10). There is yet no survival prediction model for older TGCT patients. Therefore, it is critical to develop an accurate model to estimate the prognosis of older TGCT patients. Comorbidity and age have a considerable association, which is a competing cause of death in older cancer patients (11, 12). This means that older patients are substantially more likely than younger patients to die from causes other than the target outcomes (13). When evaluating the prognosis of this aged group, competing causes of death should be addressed. In cases where competing risks are present, the naive application of Kaplan–Meier method and standard Cox regression overestimates the proportion of cancer-specific death (CSD) and may result in erroneous risk stratification (14, 15). As a result, competing risk methods are necessary for accurately estimating risk for disease in the older people.

The nomogram is a convenient prognostic tool for estimating survival outcomes that can assist doctors in making personalized decisions for patients through an intuitive graphical model (16). We used the National Cancer Institute’s Surveillance, Epidemiology, and End Results (SEER) database to collect information on older TGCT patients for competing risk analysis (17, 18). A competing risk nomogram was also developed to explore the prognostic factors associated with CSD in older TGCT patients. Based on these characteristics, we can predict the probability of CSD in patients and provide a theoretical basis for clinical decision-making.

## Patients and methods

### Data source and data extraction

Male patients over 50 years of age with a diagnosis of TGCT were identified from 17 registries in the SEER database between 2004 and 2015. As we used publicly anonymized data, our study did not require ethical review or patient consent. All methods used in this study were in accordance with the published guidelines of the SEER database. The International Classification of Diseases for Oncology, Third Edition (ICD-O-3) was used to identify cases that meet the histologic type code (9060–9102) and the primary site code (C62.0, C62.1, C62.9). Exclusion criteria included patients younger than 50 years of age, patients with diagnosis confirmed only by autopsy or death certificate or lack of histological confirmation, patients who survived less than one month, and patients for whom information on age, household income, histology, tumor stage, surgery or cause of death were not available. After data selection, the final study cohort consisted of 2,751 cases with a diagnosis of TGCT over the age of 50. For construction and validation of the nomogram, we randomly assigned 2,751 patients to the training and validation cohorts according to a simple random grouping method with a split ratio of 7:3.

We determined the prognostic factors for TGCT in the older people based on demographic and clinical variables such as age at TGCT diagnosis, race, marital status, annual household income, histology, tumor size, AJCC stage, surgery (orchiectomy), radiotherapy, and chemotherapy. In the training and validation cohorts, baseline features were compared using the χ^2^ test for categorical covariates, and Mann–Whitney test for continuous covariates. We utilized the X-tile program (Yale University, New Haven, United States) to obtain the best cut-off points. Patients’ age was divided into three groups:50–57 years, 58–67 years and ≥ 68 years, tumor size was classified as ≤4.7 cm, 4.8–7.5 cm, ≥7.6 cm and unknown, and annual household income as <$60,000, $60,000–$70,000 and >$70,000. Cancer-specific death was the primary endpoint in the study, defined as death associated with progression of testicular germ cell tumors.

### Statistical analysis

We considered cause-specific death and other causes of death as two competing events. The Fine and Gray’s test was used to estimate the cumulative incidence function (CIF) and to evaluate significant differences in CIF values between groups. The proportional subdistribution hazard model was used to identify significant variables for CSD and competitive risk nomogram was constructed based on these predictors.

The discrimination of the nomogram is reflected by the concordance index (C-index) and area under the receiver operating characteristic curve (AUC). We also used calibration curves to verify the accuracy of the nomogram. Decision curve analysis (DCA) was used to assess the net benefit at different risk thresholds to evaluate the clinical utility of the nomogram. Based on the receiver operating characteristic curve (ROC) cutoff value of the total score in the nomogram, patients were categorized into low-risk and high-risk groups. The log-rank test and Kaplan–Meier curves were used to determine differences in survival between groups. The CIF curve was used to visualize the probability of death. All statistical analyses were performed using R software version 4.3.1. A two-sided *p* value <0.05 was considered statistically significant.

## Results

### Patients baseline characteristics

As shown in Table 1, there were no statistical differences in demographic and clinical characteristics between the two subgroups (all *p* > 0.05). Table 2 summarizes the demographic and clinical characteristics of the 2,751 eligible older TGCT patients. Across the cohort, the fewest patients were aged 68 years or older (10.0%), with a majority aged 50–54 years (44.4%) and 55–67 years (45.6%). The dominant population was white (92.3%) and married (64.4%). For most, annual household income was over $70,000 (44.3%). Tumor size was usually less than 5.4 cm (62.1%). Patients were often diagnosed at an early stage (78.1%) and SGCT was the preferred histologic type (81.5%). Surgical treatment was predominant (97.0%), with 743 (27.0%) and 791 (28.8%) patients receiving radiotherapy and chemotherapy, respectively.

**Table 1:** Baseline clinicopathologic characteristics and treatment experience.

	All cohorts [*n* = 2,751]	Training cohort [*n* = 1,925]	Validation cohort [*n* = 826]	*p* value
Age (years)				0.629
Mean	57	57	57	
Standard deviation	7.3	7.2	7.6	
Race				0.588
White	2,539	1,783	756	
Black	74	49	25	
Other	102	71	31	
Unknown	36	22	14	
Marital status				0.781
Married	1,772	1,248	524	
Not married	819	566	253	
Unknown	160	111	49	
Household income				0.115
<$60,000	679	475	204	
$60,000–$70,000	854	619	235	
>$70,000	1,218	831	387	
Histology				0.679
SGCT	2,241	1,572	669	
NGCT	510	353	157	
Tumor size (cm)				0.561
≤5.4	1,710	1,186	524	
5.5–7.9	519	365	154	
≥8.0	318	233	85	
Unknown	204	141	63	
AJCC stage				0.824
I	2,148	1,497	651	
II	292	208	84	
III	311	220	91	
Surgery				0.217
No	83	53	30	
Yes	2,668	1,872	796	
Radiotherapy				0.187
No/unknown	2,008	1,391	617	
Yes	743	534	209	
Chemotherapy				0.613
No/unknown	1,960	1,366	594	
Yes	791	559	232	

**Table 2:** Three-, five-, and ten-year cumulative incidences of death in older patients with TGCT.

Characteristics	*N*	%	Event	%	Cancer-specific death (%)				Death from other causes (%)			
					3-year	5-year	10-year	*p*	3-year	5-year	10-year	*p*
Total	2,751		510		4.4	5.0	6.1		3.8	6.2	13.1	
Age (years)								0.004				<0.001
50–54	1,221	44.4	144	28.2	3.5	3.6	4.4		1.9	3.0	7.2	
55–67	1,255	45.6	238	46.7	5.0	5.9	7.0		4.2	6.6	11.6	
≥68	275	10.0	128	25.1	5.8	7.5	9.4		10.7	19.2	46.0	
Race								0.242				0.174
White	2,539	92.3	470	92.2	4.4	5.1	6.1		3.6	6.1	13.0	
Black	74	2.7	17	3.3	5.4	6.8	10.1		6.8	6.8	16.3	
Other	102	3.7	22	4.3	4.9	4.9	4.9		7.9	11.1	17.1	
Unknown	36	1.3	1	0.2	0	0	0		0	0	0	
Marital status								<0.001				<0.001
Married	1,772	64.4	261	51.2	3.1	3.7	4.4		2.7	4.8	10.7	
Not married	819	29.8	230	45.1	7.7	8.4	10.3		6.3	9.0	18.9	
Unknown	160	5.8	19	3.7	2.6	3.3	3.3		3.2	8.0	10.1	
Household income								<0.001				0.031
<$60,000	679	24.7	152	29.8	6.8	8.4	10.8		4.1	7.0	13.5	
$60,000–$70,000	854	31.0	174	34.1	4.4	4.9	5.7		4.5	7.0	15.0	
>$70,000	1,218	44.3	184	36.1	3.1	3.3	3.8		3.1	5.3	11.6	
Histology								<0.001				0.317
SGCT	2,241	81.5	382	74.9	2.6	3.2	4.1		3.7	6.4	13.5	
NGCT	510	18.5	128	25.1	12.2	13.1	14.6		4.2	5.6	11.1	
Tumor size (cm)								<0.001				<0.001
≤5.4	1,710	62.1	233	45.7	2.3	2.7	3.4		2.2	4.2	10.6	
5.5–7.9	519	18.9	115	22.6	3.3	4.3	6.4		6.2	10.1	16.9	
≥8.0	318	11.6	96	18.8	11.1	12.8	13.6		7.6	10.0	19.1	
Unknown	204	7.4	66	12.9	14.8	14.8	16.0		4.9	7.5	15.5	
AJCC stage								<0.001				0.464
I	2,148	78.1	330	64.7	1.1	1.7	2.7		3.4	6.2	12.9	
II	292	10.6	43	8.4	4.1	4.1	4.7		2.8	3.9	12.0	
III	311	11.3	137	26.9	27.5	28.9	30.9		7.8	8.8	16.3	
Surgery								<0.001				0.812
No	83	3.0	38	7.5	31.3	32.6	34.2		6.0	6.0	13.5	
Yes	2,668	97.0	472	92.5	3.6	4.2	5.2		3.7	6.2	13.1	
Radiotherapy								0.021				0.692
No/unknown	2,008	73.0	387	71.7	5.2	5.7	6.6		4.2	6.5	13.6	
Yes	743	27.0	153	28.3	2.4	3.3	4.5		2.8	5.5	11.9	
Chemotherapy								<0.001				0.187
No/unknown	1,960	71.2	350	64.8	1.5	2.1	3.1		3.5	6.4	13.5	
Yes	791	28.8	190	35.2	11.6	12.3	13.6		4.5	5.8	12.3	

TGCT, testicular germ cell tumor; SGCT, seminomatous germ cell tumor; NSGCT, nonseminomatous germ cell tumor; AJCC, American Joint Committee on Cancer.

**Figure 1:**
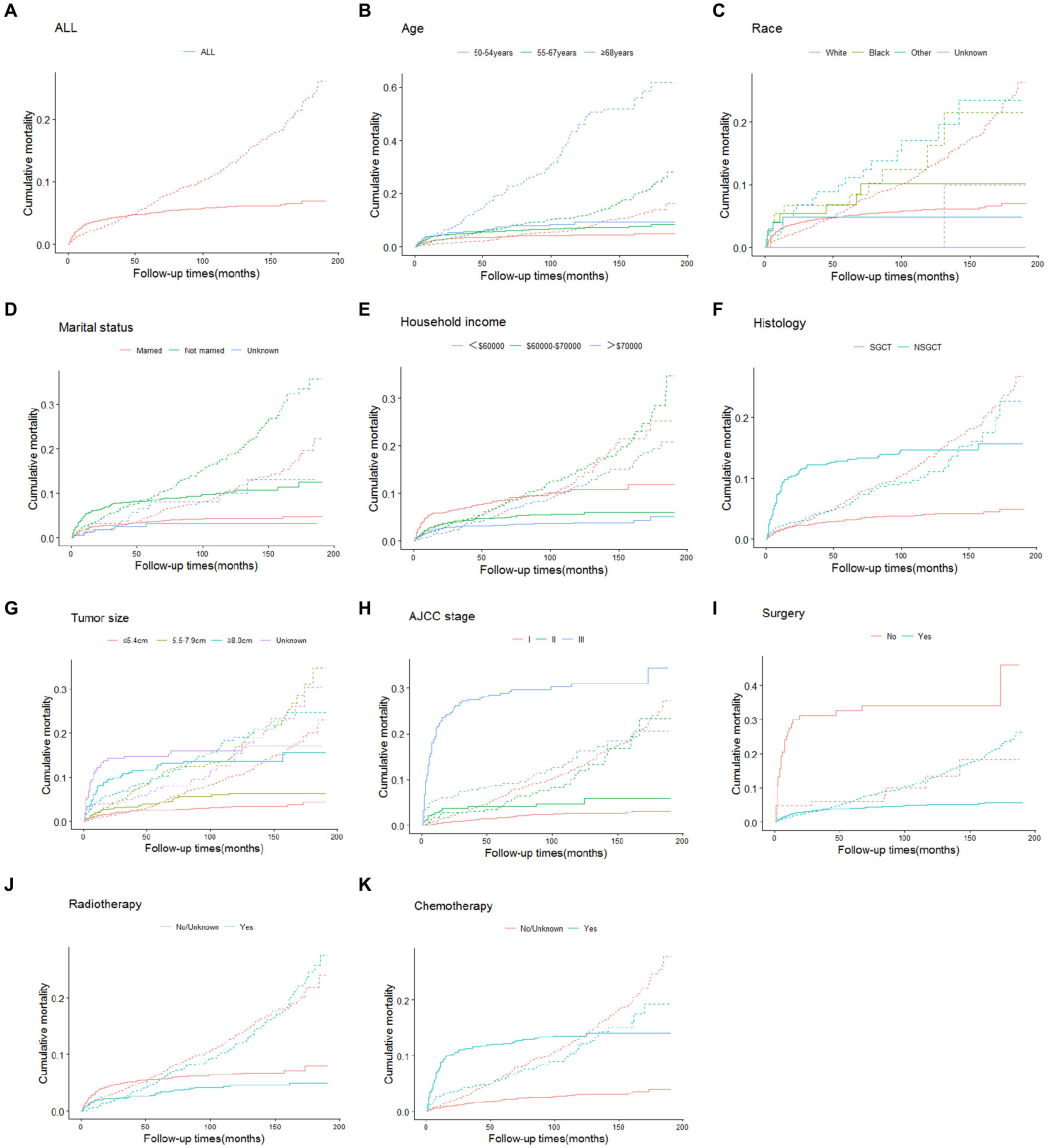
Cumulative incidence estimates of death according to patient characteristics (solid line indicates cause-specific death; dotted line indicates other cause of death): all **(A)**; age **(B)**; race **(C)**; marital status **(D)**; household income **(E)**; histology **(F)**; tumor size **(G)**; AJCC stage **(H)**; surgery **(I)**; radiotherapy **(J)**; chemotherapy **(K)**. SGCT seminomatous germ cell tumor, NSGCT nonseminomatous germ cell tumor, AJCC American Joint Committee on Cancer.

The cumulative incidences of cause-specific and other causes of death at 3-, 5-, and 10-year by age at TGCT diagnosis, race, marital status, annual household income, histology, tumor size, stage and treatment are shown in Table 2. The 3-, 5-, and 10-year cumulative incidence for cause-specific of death were 4.4, 5.0 and 6.1%, respectively, among TGCT, and 3.8, 6.2 and 13.1%, respectively, for other causes of death. As shown in Figure 1, the CIF curves indicated that patients who were older, unmarried, at lower annual household income, or with larger tumor size were at risk of dying from TCGT and competing events. Patients with histologic type NSGCT, advanced stage, no surgery or radiotherapy, and who received chemotherapy had an increased cumulative mortality from TCGT, independent of competing causes. There were no statistically significant differences in cancer-specific mortality among races.

### Independent predictors of older patients with TGCT

The subdistribution risk ratios (HRs) of the competing risk model for cause-specific mortality in older TGCT are presented in Table 3. Getting older was associated with an increased probability of dying from TGCT. Advanced stage (stage III) was a strong predictor of cause-specific mortality. Patients with annual household income >$70,000 and those who had undergone surgery experienced a reduction in cause-specific mortality, with HR of 0.45 (95% confidence interval [CI] 0.28–0.71) and 0.47 (95% CI 0.26–0.87), respectively. Being unmarried was associated with an increased risk of cause-specific mortality, with an HR of 1.88 (95% CI 1.26–2.79). In addition, histology as NSGCT and larger tumor size were linked to poorer outcomes. Radiotherapy or chemotherapy did not predict the probability of cause-specific mortality.

**Table 3:** Proportional subdistribution hazard models of probabilities of CSD for older patients with TGCT in the training cohort.

Characteristics	HR (95% CI)	*p*
Age (years)	1.03 (1.01–1.06)	0.002
Marital status		
Not married	1.88 (1.26–2.79)	0.002
Unknown	0.71 (0.22–2.31)	0.570
Household income		
$60,000–$70,000	0.64 (0.41–1.00)	0.051
>$70,000	0.45 (0.28–0.71)	<0.001
Histology		
NGCT	2.46 (1.59–3.82)	<0.001
Tumor size (cm)		
5.5–7.9	1.57 (0.92–2.68)	0.098
≥8.0	2.06 (1.25–3.40)	0.005
Unknown	1.93 (1.04–3.58)	0.036
AJCC stage		
II	1.37 (0.71–2.68)	0.35
III	5.85 (3.42–10.00)	<0.001
Surgery	0.47 (0.26–0.87)	0.016
Radiotherapy	1.75 (0.97–3.17)	0.063
Chemotherapy	1.58 (0.91–2.69)	0.100

CSD, cause-specific death; TGCT, testicular germ cell tumor; SGCT, seminomatous germ cell tumor; NSGCT, nonseminomatous germ cell tumor; AJCC, American Joint Committee on Cancer.

### Construction and validation of the nomogram

**Figure 2:**
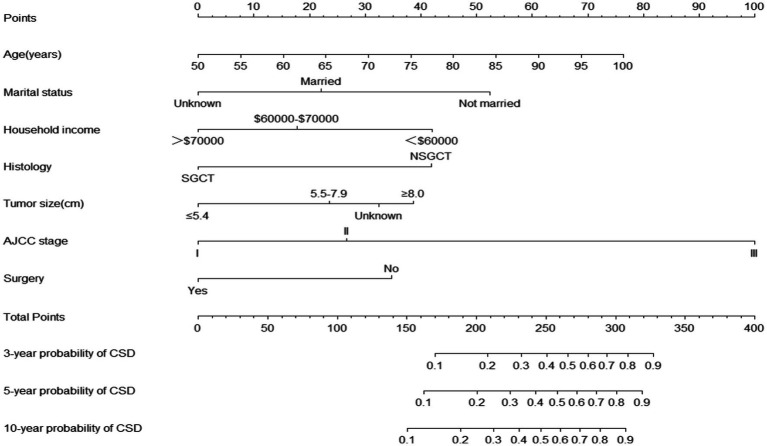
Nomogram predicting the probabilities of 3-, 5-, and 10-year cancer-specific death. SGCT, seminomatous germ cell tumor; NSGCT, nonseminomatous germ cell tumor; AJCC, American Joint Committee on Cancer; CSD, cause-specific death.

The nomogram for predicting the probability of CSD in older TGCT patients based on the Fine and Gray’s model is shown in Figure 2. For each patient, the values of the different variables are first located in the corresponding rows, and then a vertical line is drawn pointing to the “Points” row to obtain the corresponding scores. By adding these scores together, a total score is obtained and a vertical line is drawn from the total points row to obtain the probability of CSD at 3-, 5-, and 10-year.

**Figure 3:**
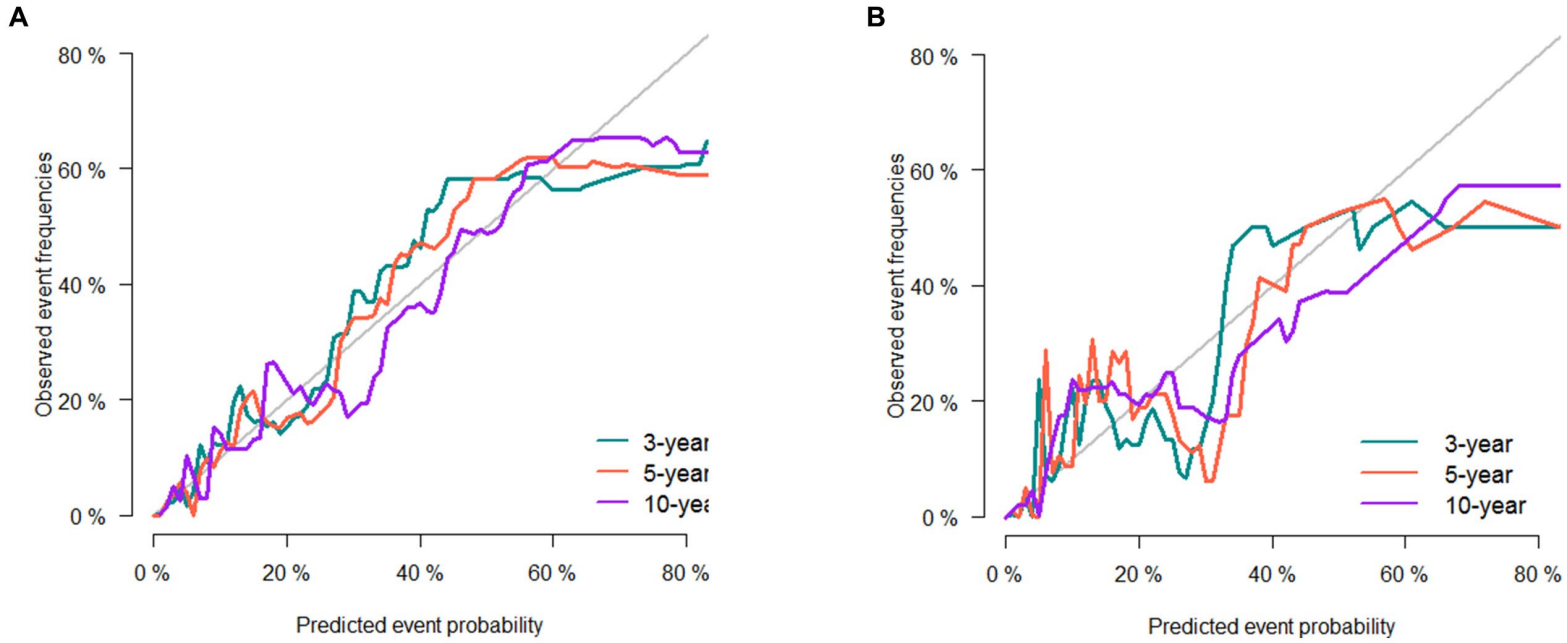
Calibration curve of the nomogram in the training set **(A)** and validation set **(B)**. The horizontal axis is the predicted value in the nomogram, and the vertical axis is the observed value.

**Figure 4:**
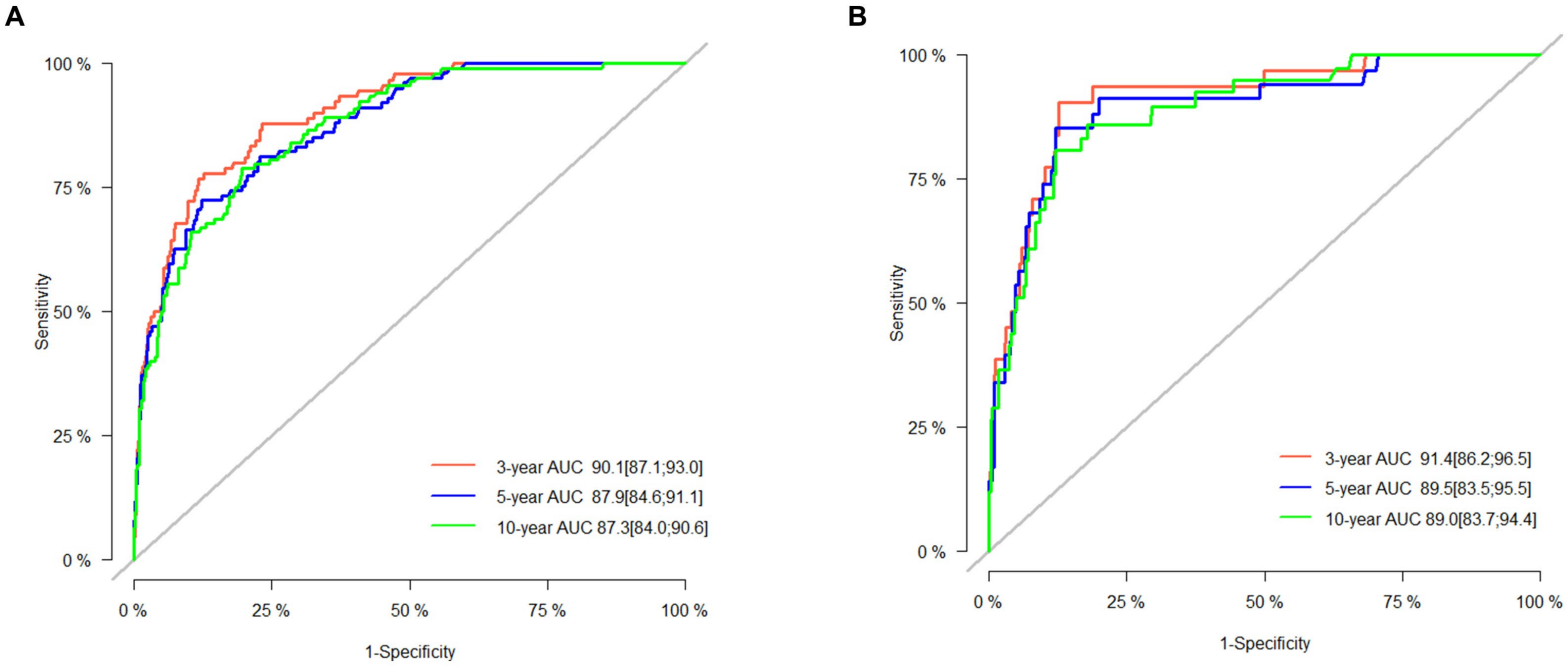
AUC for predicting 3-, 5-, and 10-year CSS in the training set **(A)** and validation set **(B)**.

**Figure 5:**
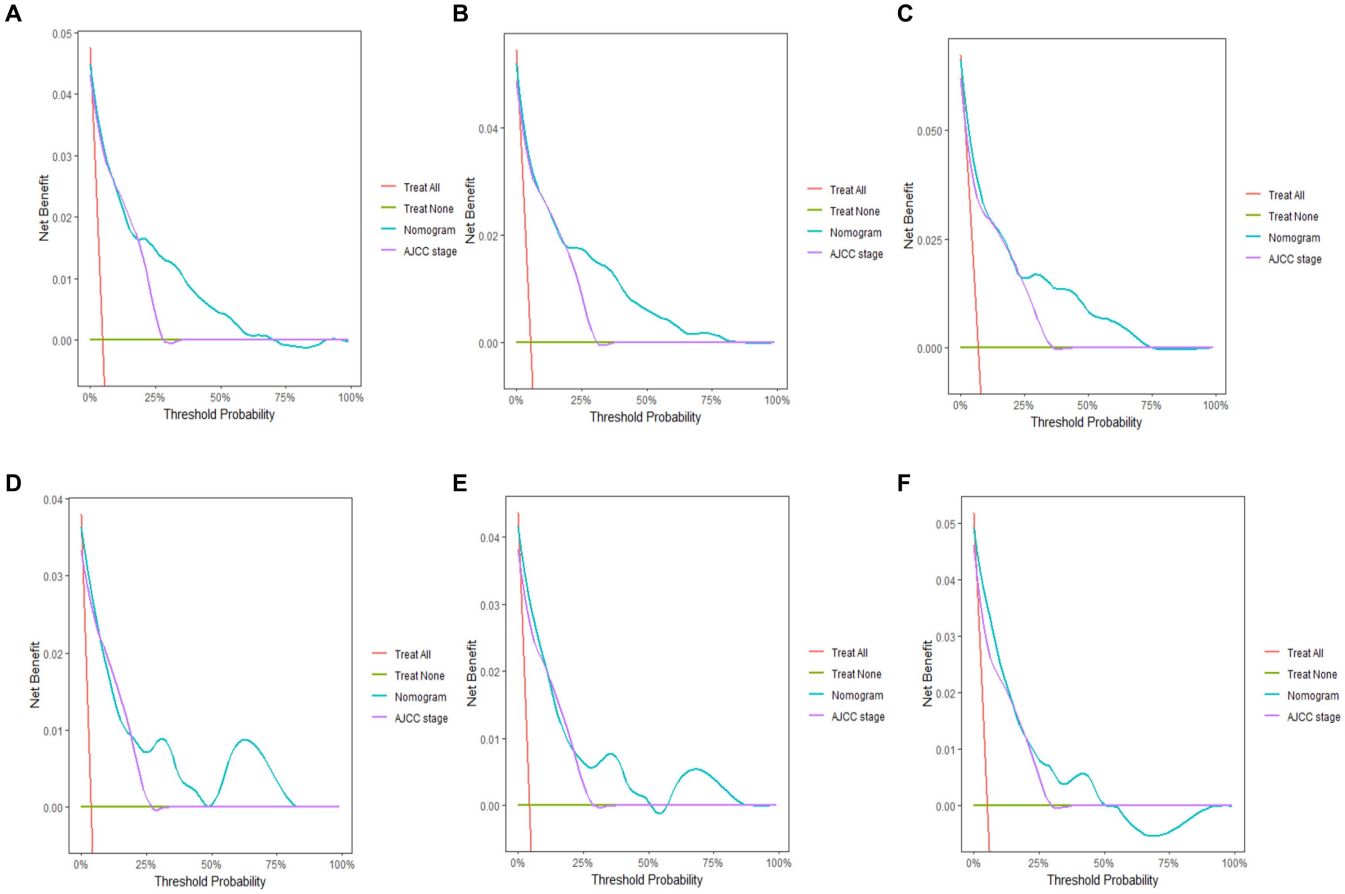
Decision curve analysis of the nomogram. Y-axis represents a net benefit and x-axis represents threshold probability. The green line means no patient died and the red line means all patients died. **(A)** 3-year survival benefit for the training cohort. **(B)** 5-year survival benefit for the training cohort. **(C)** 10-year survival benefit for the training cohort. **(D)** 3-year survival benefit for the validation cohort. **(E)** 5-year survival benefit for the validation cohort. **(F)** 10-year survival benefit for the validation cohort.

The 3-, 5-, and 10-year C-indexes for the nomogram were 0.89, 0.87 and 0.85 in the training cohort and 0.91, 0.89 and 0.88 in the validation cohort, respectively, which suggested excellent model differentiation. In Figure 3, the calibration curves were close to the 45-degree diagonal, indicating that the developed nomogram was well-calibrated (with good consistency between observed and predicted probability of death). As shown in Figure 4, in the training cohort, the AUC values were 90.1, 87.9 and87.3 at 3-, 5-, and 10-year, respectively, while in the validation cohort, the AUC values were 91.4, 89.5 and 89.0 at 3-, 5-, and 10-year, respectively, indicating that the predictive model was well discriminated. DCA curves showed that the clinical value of the nomogram was superior to the AJCC staging system in both the training and validation groups (Figures 5A–F).

**Figure 6:**
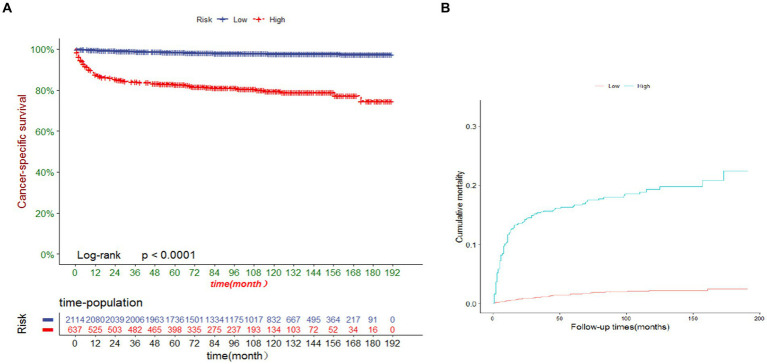
**(A)** Cancer-specific survival and **(B)** cumulative incidence of cause-specific death for patients in the low-risk and high-risk groups.

We developed a risk stratification system using the ROC cutoff value to categorize patients into two groups: low risk (total score ≤ 105.7) and high risk (total score > 105.7). As depicted in Figure 6, the cancer-specific survival (CSS) was lower in the high-risk group than in the low-risk group, and the probability of CSD was higher in the high-risk group than in the low-risk group (both *p* < 0.0001). The 3-, 5-, and 10-year CSS in the high-risk group were 98.9, 98.4 and 97.8%, respectively, compared with 83.9, 82.8 and 79.5% in the low-risk group. The 3-, 5-, and 10-year CSD for the high-risk group were 15.5, 16.5 and 19.3%, respectively, and 1.1, 1.6 and 2.2% for the low-risk group.

## Discussion

There is no consensus on the age definition of older TCGT patients. However, the relative survival of TGCT patients aged ≥50 years was significantly lower than that of patients aged <50 years and was characterized by distinct clinical features in terms of histologic type and stage (19, 20). Consequently, we defined the age criterion for older patients in this study as 50 years. We calculated the CIF for cause-specific and competitive risk of death in older TGCT patients. In the case of competing risks, the CIF provides an unbiased estimate of the probability of a certain event (14). To the best of our knowledge, this is the first study to attempt to build a nomogram for predicting CSD in older TGCT using a proportional subdistribution risk approach based on population data. The nomogram can be immensely helpful for clinicians in caring for older patients with TGCT to discuss treatment modalities and prognosticate. After obtaining basic information about the patient such as age, marital status, annual household income, and diagnostic information such as histologic type, tumor size, and AJCC stage, the treatment can be discussed based on the nomogram. After the patient has undergone treatment, the physician can again predict the patient’s prognosis based on the nomogram.

In our competing risk model, age, marital status, annual household income, histology, AJCC stage, tumor size, and surgery had a significant effect on CSD in older TGCT patients. Age is associated with the prognosis of most malignant tumors. Many studies have shown that increasing age is linked with recurrence, metastasis, and mortality in TGCT. According to a recent report by the International Germ Cell Cancer Cooperative Group (IGCCG-Update Consortium), the risk of progression increases by 25% for each decade of life expectancy in metastatic NSGCT (21). In comparison to younger patients, Fosså SD et al. discovered a significant twofold increase in TGCT-specific mortality in men over 40 years of age (8). Similarly, data from the Danish Population-Based Cancer Registry indicated that age could be a new prognostic factor for TGCT recurrence and mortality. Aging for 10 years increases the risk of death due to TGCT by 1.3-fold (22). In this study, socioeconomic-related factors, such as annual household income, was also a risk indicator for patient mortality. Consistently, existing publications have shown that TGCT patients with lower socioeconomic status are more likely to have advanced disease as well as higher overall and cancer-specific mortality compared to cases with higher socioeconomic status (8, 10, 23). In addition to annual household income, marital status was employed as one of the predictors for the CSD in this study. Previous studies have shown that unmarried men had two- to three-fold excess mortality compared to married men (8, 24). Potential mechanisms may include earlier detection, improved compliance, and more social support for married TGCT patients (24).

Our study demonstrated that staging is a significant prognostic variable in older TGCT patients, which is consistent with a previous retrospective report from a large cohort. Patients with advanced stage (stage III) tend to have a higher probability of CSD. The previous study showed that TCGT patients with AJCC stage III exhibited the highest 5-year CSD (SGCT: stage I:0.4; stage II:3.4; stage III:11.4%; *p* < 0.01; NSGCTT: stage I:1.6; stage II:2.5; stage III 22.2%; *p* < 0.001) (25). Meanwhile, we discovered that patients with a histologic type of NSGCT had a worse prognosis compared to SGCT. Patients with TGCT in the presence of distant metastasis predominantly present with NSGCT (21, 26). The poor outcome of TGCT is mainly driven by distant metastasis. The presence of lung metastasis implies a 62% increased risk of progression compared to NSGCT patients without lung metastasis. Patients who had non-pulmonary visceral metastases to the bone, liver, or brain had a 5-year progression-free survival of less than 78% (21). Furthermore, those patients carrying brain metastasis demonstrated the worst survival rates, with more than half of them experiencing disease progression and death within one year of confirmed intracranial involvement (27).

In our study, tumor size has also been identified as an independent predictor of TGCT in older adults. Larger tumor size tends to suggest a poor prognosis. Several investigators have found that tumor size is related with recurrence in clinical stage I NSGCT cohorts, with significant thresholds of 4 cm and 5 cm (28, 29). A study including 219 patients with NSGCT demonstrated that tumor size was a strong predictor of metastatic disease at the time of diagnosis, with a significant threshold of 6 cm (30). However, several other studies have not consistently shown a substantial association (31, 32). Different from NSGCT, tumor size in SGCT is a well-known prognostic factor for advanced clinical stage and metastatic disease.

Radical orchiectomy is the primary treatment for most patients with TGCT (33). After evaluating the relationship between age and outcomes in colon, lung, hepatobiliary, and head and neck cancers, several researchers concluded that surgery remains the best treatment for solid tumors and the actual age itself should not be a determining factor in therapeutic decisions (34). Our study confirmed that surgical treatment was a protective factor in older TGCT patients, with a remarkably better prognosis than the non-surgical treatment group. Currently, there is still no consensus on adjuvant treatment options TGCT in the older people after orchiectomy. Our data showed that radiotherapy improved patients’ survival, whereas chemotherapy had the reverse result. However, after adjusting for confounders, they could not be used as prognostic predictors. Historically, adjuvant radiotherapy or chemotherapy aimed to reduce the probability of recurrence in patients with TGCT for the sake of improving survival (34). Nevertheless, there is evidence that radiotherapy or chemotherapy has adverse effects on TGCT. A previous study revealed that radiotherapy or chemotherapy increased the risk of developing a second malignancy by 2.6 times after radiotherapy and 2.1 times after chemotherapy (35). Furthermore, a recent study reported that patients treated with cisplatin, bleomycin, and etoposide alone had a 5.7-fold higher risk of cardiovascular disease compared with patients who received surgery only (36). Another report based on a cohort of 453 male patients with SGCT who underwent orchiectomy and radiotherapy showed a standardized mortality rate (SMR) of 1.59 (99% CI 1.21–2.04), with a significant increase after 15 years of radiotherapy (37). Especially in the older people, toxicity may be generated at lower doses of radiation due to altered organ function and the presence of concomitant diseases (38). On the contrary, some other studies have shown the opposite results (39, 40). Since testicular cancer is rare in the older people, there are little data on the clinical characteristics and prognosis of patients with TGCT after the age of 50 years. Therefore, prospective studies should be designed specifically for the older people in order to develop optimal treatment regimens for them.

TGCT is a rare malignancy in the older people, making it challenging to assess its prognosis. The SEER database is able to provide a sufficiently large and representative sample. We established this nomogram using the SEER database and verified its validity. Taken together, our nomogram may be a useful method for assessing the prognosis of older TGCT patients. In addition, the risk stratification of our nomogram helps to identify high-risk groups, thereby providing them with appropriate clinical guidance.

However, there are some deficiencies in this study. First, the SEER database lacks other crucial variables such as lifestyle, genetic background, and environmental factors that may influence the prognosis of older TGCT patients. Second, as a retrospective study, selection bias may be inevitable. For example, the subjects of this study were mainly focused on high-income groups and Caucasians, which may have reduced the breadth of our results. Instead, we incorporated important parameters such as age, stage and treatment to ensure that the results were not considerably biased. Third, we were unable to obtain detailed information about cancer recurrence and treatment, which hindered further analysis. Finally, although our model exhibited excellent performance in predicting the probability of CSD in older TGCT patients, prospective validation in a multicenter study is needed to confirm the accuracy of the model.

## Conclusion

In summary, this study establishes a valuable nomogram for the prediction of CSD in older TGCT patients, which may provide a meaningful reference for the treatment of this special population.

## Data availability statement

The original contributions presented in the study are included in the article/supplementary material, further inquiries can be directed to the corresponding authors.

## Ethics statement

The studies involving humans were approved by the data of this study is obtained from the SEER database. The patients’ data is public and anonymous, so this study does not require ethical approval and informed consent. The studies were conducted in accordance with the local legislation and institutional requirements. The participants provided their written informed consent to participate in this study. Written informed consent was obtained from the individual(s) for the publication of any potentially identifiable images or data included in this article.

## Author contributions

XW: Conceptualization, Data curation, Formal analysis, Investigation, Methodology, Resources, Software, Validation, Visualization, Writing – original draft. MZ: Writing – original draft. JL: Supervision, Writing – review & editing. LC: Supervision, Writing – review & editing.
